# In Response

**DOI:** 10.4269/ajtmh.2011.11-0114b

**Published:** 2011-06-01

**Authors:** Dionne Bezerra Rolim, Luciano Pamplona de Góes Cavalcanti, Timothy J. J. Inglis, Aparecida Tiemi Nagao-Dias

**Affiliations:** University of Fortaleza, Brazil; E-mail: dionnerolim@gmail.com; Christus Faculdade of Fortaleza and Department of Community Health; Federal University of Ceará, Brazil; E-mail: Pamplona.luciano@gmail.com; Division of Microbiology and Infectious Diseases; PathWest Laboratory Medicine WA, QEIIMedical Centre, Nedlands, Perth, Western Australia, Australia; E-mail: tim.inglis@health.wa.gov.au; Department of Clinical Analysis and Toxicology; Federal University of Ceará, Brazil; E-mails: tiemindi@yahoo.com.br

Dear Sir:

The concerns that are raised by Peacock and others^1^ about caution against the use of an unvalidated enzyme-linked immunosorbent assay (ELISA) are critically important. The aim of our work was to perform a serological study in some regions of Ceará, Brazil, where culture-confirmed cases of melioidosis had been reported^2^ and where bacteria has been isolated from the soil.^3^ We thank Dr. Peacock for his kind letter^1^ and agree with his assessment that there is a risk of false positive results with any serological method. We considered this possibility in our data and mentioned it in our discussion.^4^ We remind the authors that we used a culture filtrate of *B. pseudomallei*, as recommended by others.^5^

We just reinforce that our work aimed to draw attention to the need to establish an epidemiological surveillance for cases of melioidosis in northeastern Brazil, where the disease is still unknown. We agree with the authors that the indirect hemagglutination assay (IHA) method is a valid and widely used technique, and endorse their call for an international serological standard. In the meantime, we intend to establish the IHA method as an interim measure to allow comparison with results from other centers.
